# Unusual Presentation of Extrapulmonary Tuberculosis as Laryngeal Mass in an Atypical Patient

**DOI:** 10.1155/carm/9912317

**Published:** 2024-12-13

**Authors:** Rohan Dipesh Agarwal, Mark David Marino, Matthew Joseph Whalen, Ronald J. Walker

**Affiliations:** Department of Otolaryngology, Head and Neck Surgery, Saint Louis University School of Medicine, St Louis, Missouri, USA

## Abstract

Spread of Mycobacterium tuberculosis (MTB) to the larynx is exceedingly rare and can be obscured by more common conditions such as laryngeal cancer or oropharyngeal candidiasis, complicating an accurate diagnosis. Risk factors for chronic laryngeal disease, such as smoking and toxin exposure, place TB infection comparatively lower for consideration on a physician's differential. However, identifying these lesions is crucial from a medical and public health perspective to prevent community spread. We report the case of a 60-year-old male who presented with dysphonia, pharyngitis, aphasia, and significant unintentional weight loss. X-rays demonstrated focal opacities in the upper lung lobes. Laryngoscope biopsy revealed caseating granulomas and a positive culture for MTB. Laryngeal TB presents as a suspicious throat mass with nonspecific symptoms and should be thoroughly investigated by clinicians.

## 1. Background

Tuberculosis (TB) is a global infectious disease with diverse manifestations, necessitating prompt and accurate diagnosis for effective treatment. In 2022, there were 8,300 TB cases reported in the United States, with close to 20% being extrapulmonary, with the head and neck comprising 10% of all patients, and disease of the larynx being a rare occurrence [1–3]. Over the past 30 years, case studies have reported TB of the larynx most frequently in females under 40 [4]. Compared to its classical presentations, extrapulmonary TB requires more invasive workup using fine-needle aspiration, cytology, and direct biopsy. The following story is an example of an unusual presentation of extrapulmonary TB that echoes the importance of considering a broad differential.

## 2. Case Presentation

We present an elderly male (patient) with past medical history significant for chronic obstructive pulmonary disease (COPD), 22.5 pack/year smoking history, and occupational exposure to inhaled dust and silica.

**Figure 1:**
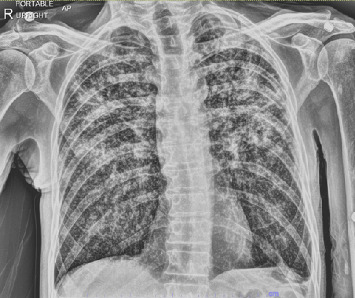
Initial single-view PA chest X-ray.

The patient first presented to the emergency department in late 2022 for cough, sore throat, and difficulty swallowing. Chest X-ray (Figure 1) and computed tomography (CT) imaging revealed severe bilateral diffuse pulmonary infiltrates and multifocal consolidation in the upper lobes. Results were indicative of sepsis secondary to bilateral pneumonia. The patient was then treated with antibiotics, steroids, and oxygen therapy. He continued to have sore throat and dysphagia following hospital discharge and was evaluated by an otolaryngologist a few weeks later. At follow-up, the patient was diagnosed with oropharyngeal and laryngeal fungal infection and treated with Diflucan and nystatin, which provided temporary relief. Despite the treatment, the patient's health continued to deteriorate with worsening dysphagia, sore throat, dysphonia, dyspnea, and a 30-pound weight loss over two months.

At the two-month follow-up, a thorough physical exam was suggestive of a throat mass with mucus production. He underwent fiberoptic laryngoscopy (CPT), which uncovered diffuse mucosal changes with a thick mucopurulent material. The laryngoscopy showed irritation and inflammation with mucoid drainage along the entire endolarynx and laryngeal surface of the epiglottis. Consequently, the patient underwent direct laryngoscopy (MicroFrance) with gastric-tube placement and biopsy.

**Figure 2:**
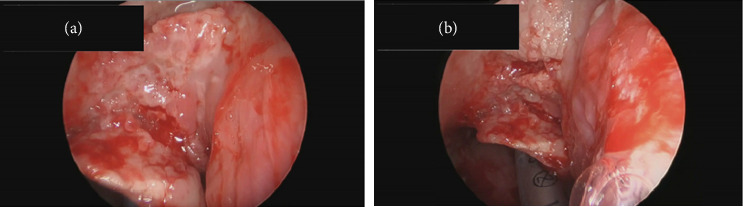
(a) Direct laryngoscopy exophytic mass, close-up view. (b) Direct laryngoscopy exophytic mass, normal view.

**Figure 3:**
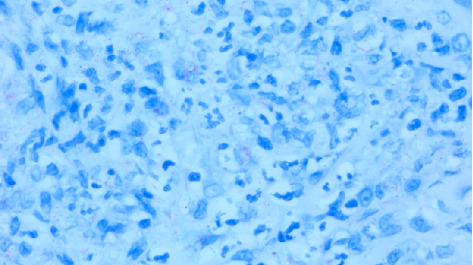
A 10x-magnification of squamous mucosa epiglottis with caseating granuloma.

**Figure 4:**
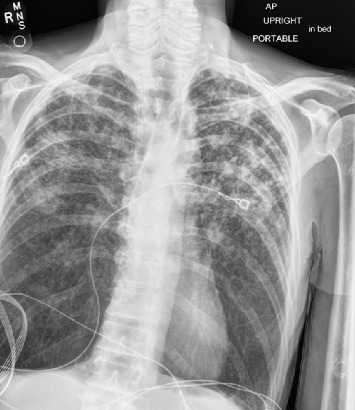
Repeat single-view PA chest X-ray.

**Figure 5:**
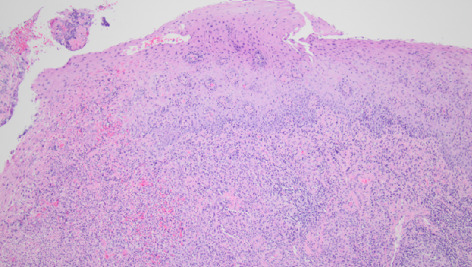
AFB positive smear and culture of laryngeal biopsy specimen.

The direct laryngoscopy (Figures 2(a) and 2(b)) demonstrated the abnormal tissue on the left larynx, and the lingual surface of the epiglottis and vallecula. Granular exophytic lesions on the left arytenoid left larynx appeared without discrete mass, ulceration, or purulence. A biopsy was performed of the left arytenoid, epiglottis, and pre-epiglottic area along with Gram stain, fungal culture, and acid-fast bacilli (AFB) smear with culture. Pathology of the specimens revealed marked necrotizing acute inflammation and granulation tissue negative for malignancy (Figure 3). Postoperatively, the patient developed a fever to 102.5°F (39.2°C) with increasing oxygen requirement. A repeat chest X-ray (Figure 4) revealed bilateral upper lobe opacities mirroring the pneumonia on the initial ED X-ray. Ultimately, the laryngeal biopsy resulted positive on AFB smear and Mycobacterium TB (MTB) on culture (Figure 5). These results correlated to positive sputum AFB smear with culture and nucleic acid amplification testing (NAAT), confirming the diagnosis of active laryngeal and pulmonary TB. Notably though, blood cultures were negative for TB.

The patient started treatment for TB with rifampin, isoniazid, pyrazinamide, and ethambutol (RIPE) therapy, which were tolerated well during hospitalization. The patient's condition improved to his prior baseline, and he was eventually discharged from the hospital. He was closely followed by the State Department of Health and was adherent to his treatment regimen for 60 days, until being lost to follow-up.

## 3. Discussion

Laryngeal TB remains difficult to diagnose due to its uncommon occurrence. One meta-analysis found laryngeal TB accounting for 8.7% of extrapulmonary TB infections in the head and neck [4]. Though laryngeal TB shares several common risk factors to classic TB, such as human immunodeficiency virus positive status and immigration from foreign endemic areas, relying on epidemiology alone can also obscure the diagnosis of the disease in patients such as our case [1, 2].

The disease lacks many classic characteristics of pulmonary TB, with its mass-like appearance enabling it to masquerade as malignancy. In previous case series and reports of patients with laryngeal TB, the most common presenting complaint is hoarseness. These lesions typically involve the true vocal folds, arytenoid cartilage, and epiglottis on laryngoscopy [5–7]. These patients were mostly from Asia or immigrants to the United States, differing from our patient's demographic. In our case, the patient had a known previous medical history of COPD, candidiasis, and multifocal pneumonia supported by medical imaging. Furthermore, a 22.5 pack/year smoking history and occupational plastic silica exposure contributed to a differential that placed fungal infection and cancer higher on the list.

This case demonstrates that laryngeal TB should be considered as a source of throat disease, even in patients from developed countries with no known sick contact exposure, travel history, or other pertinent risk factors. Though TB has classically been associated with the developing world, there has been a shift in TB demographics over the last three decades partly due to the HIV/AIDS epidemic and increasing utilization of immunosuppressive therapies [2]. One case in the United Kingdom involving a female with the same age range as our patient presented with a similar presentation and initial complaint as our patient [8]. However, her history of long-term steroid therapy for nephrotic syndrome was more telling of a potential reactivated infectious disease such as TB, whereas our patient was not on any chronic steroid or immunosuppressive regimen.

It is also important to consider the role COPD can play in both masking and potentially triggering TB infection. COPD increases the risk of pulmonary infections due to airway inflammation, impaired, debris clearance, and alveolar immune system dysfunction. Therefore, inflammation and consolidation in multifocal pneumonia secondary to COPD could have masked TB on initial X-ray. One study found COPD individuals having 2.4x greater risk of developing TB than non-COPD individuals [9]. Given this patient's presentation and pathology results, it is difficult to ascertain whether laryngeal TB arose from a reactivated pulmonary infection or if TB infected the larynx as a primary site before spreading inferiorly to the lungs, given the fact that the patient did not have any previous recorded interferon-release assay or tuberculin skin testing.

More research is needed to adequately understand laryngeal TB, especially in developed nations. Contrasting tuberculoid lesions of the larynx from malignancy requires more invasive diagnostics, as presenting symptoms can mimic one another. Physicians must thoroughly investigate atypical lesions and patient presentations, rather than relying on pretest probability and imaging alone, to capture laryngeal TB early. Current Infectious Diseases Society of America guidelines for diagnosing extrapulmonary TB in suspected specimens include AFB smear microscopy, MTB culture, NAAT, and tissue pathology [10]. Tissue biopsy of the larynx is crucial in diagnosing patients, as imaging and sputum culture alone cannot definitively rule out other diseases in patients presenting with symptoms concerning for throat mass.

## 4. Conclusion

A diagnosis of laryngeal TB can be easily overlooked, and it poses a serious public health risk. Disease presentation is complicated by nonspecific symptoms and other pulmonary diseases based on history alone. Therefore, clinicians must diligently work up suspicious throat masses in symptomatic individuals to evaluate rare disease presentations such as laryngeal TB. Clinicians should consider ordering biopsy pathology, as well as AFB smears, and cultures for extrapulmonary specimens to rule out active laryngeal TB, especially when considering a broad differential for patients with nonspecific clinical features.

## Data Availability

Data sharing is not applicable to this article as no datasets were generated or analyzed during the current study.
